# Role of protein kinase C β and vascular endothelial growth factor receptor in malignant pleural mesothelioma: Therapeutic implications and the usefulness of *Caenorhabditis elegans* model organism

**DOI:** 10.4103/1477-3163.77274

**Published:** 2011-03-03

**Authors:** Sivakumar Loganathan, Rajani Kanteti, Shahid S. Siddiqui, Essam El-Hashani, Maria Tretiakova, Hari Vigneswaran, Gustavo Cervantes, Viswanathan Natarajan, Aliya N. Husain, Everett E. Vokes, Hedy L. Kindler, Ravi Salgia

**Affiliations:** 1 Section of Hematology/Oncology, University of Chicago, Chicago, IL 60637, USA; 2 Section of Pulmonary Medicine, Department of Medicine, University of Chicago, Chicago, IL 60637, USA; 3 Department of Pathology, University of Chicago, Chicago, IL 60637, USA; # These two authors contributed equally to the paper

**Keywords:** Enzastaurin, KRN633, malignant pleural mesothelioma, PKC-β, VEGFR-2

## Abstract

**Purpose:**

To examine the role of both protein kinase C (PKC)-β and vascular endothelial growth factor receptor (VEGFR)-2 in malignant pleural mesothelioma (MPM) using respective inhibitors, enzastaurin and KRN633.

**Materials and Methods:**

MPM cell lines, control cells, and a variety of archived MPM tumor samples were used to determine the protein expression levels of PKC-β, VEGFR-2, VEGF, and p-AKT. Effects of enzastaurin and KRN633 on phosphorylation status of key signaling molecules and viability of the mesothelioma cells were determined. The common soil nematode, *Caenorhabditis elegans*, was treated with enzastaurin to determine its suitability to screen for highly potent kinase inhibitors.

**Results:**

PKC-β1, PKC-β2 and VEGFR-2/KDR were overexpressed in MPM cell lines and MPM tumor tissues. Enzastaurin treatment resulted in significant loss in viability of VEGF induced cell proliferation; however, the effect of KRN633 was much less. Enzastaurin also dramatically decreased the phosphorylation of PKC-β, its downstream target p-AKT, and surprisingly, the upstream VEGFR-2. The combination of the two drugs at best was additive and similar results were obtained with respect to cell viability. Treatment of *C. elegans* with enzastaurin resulted in clear phenotypic changes and the worms were hypermotile with abnormal pattern and shape of eggs, suggesting altered fecundity.

**Conclusions:**

PKC-β1 and VEGFR-2 are both excellent therapeutic targets in MPM. Enzastaurin was better at killing MPM cells than KRN633 and the combination lacked synergy. In addition, we show here that *C. elegans* can be used to screen for the next generation inhibitors as treatment with enzastaurin resulted in clear phenotypic changes that could be assayed.

## BACKGROUND

Malignant pleural mesothelioma (MPM) is a devastating disease and the prognosis is relatively poor despite recent advances in chemotherapy. There is a clear and urgent requirement for the identification of novel mesothelioma targets for the development of highly efficacious therapeutics. In this paper, we have shown that both protein kinase C (PKC-β1 and -β2 isoforms) and vascular endothelial growth factor receptor (VEGFR)-2 are overexpressed in different types of mesotheliomas, thereby making them excellent combinatorial therapeutic targets. Enzastaurin, in comparison to KRN633, was highly effective in arresting key phosphorylation events and cell survival; however, the combination of the two was at best additive. As PKC appears to be the most attractive primary chemotherapeutic target, we subjected it to further study. Here, we have shown that the common soil nematode, *Caenorhabditis elegans*, can be potentially used to screen (*in vivo*) for the next generation inhibitors as treatment with enzastaurin resulted in clear phenotypic changes that could be assayed.

MPM is a highly aggressive tumor that is largely unresponsive to conventional chemotherapy or radiotherapy, and most patients die within 10–17 months of the initial diagnosis. There is no known curative modality for advanced MPM and long-term survival rate is low even with aggressive multimodality therapy.[1–2] VEGF is a critical angiogenic factor that plays a very important role in tumor growth and metastasis.[3] VEGFR, when ligated with its natural ligand VEGF, initiates a cascade of signaling events that ultimately result in tumor angiogenesis and neovascularization. An important downstream signal transducer in the VEGF/VEGFR signaling pathway is PKC-β.[4–5] PKC-β in turn activates the Mitogen-activated protein (MAP) kinase pathway which ultimately promotes neovascularization and angiogenesis.[6] The family members of PKC are multifunctional protein kinases that specifically phosphorylate serine and threonine amino acid residues in target proteins. To date, 12 isoforms of PKC have been described that differ in primary structure, tissue distribution, subcellular localization and substrate specificity.[7] The PKC-β isoform is a key component in the VEGF/VEGFR signaling cascade, and inhibiting PKC-β results in reduced tumor vascularization that ultimately leads to tumor regression. Furthermore, inhibition of angiogenesis has been shown to confer a survival benefit in a phase III trial in non-small cell lung cancer (NSCLC).[8] VEGFR-2 is thought to provide both mitogenic and survival signals, and to be the major signaling component in angiogenesis. Blockade of VEGF signaling is therefore a very attractive therapeutic target in the treatments of solid tumors. Enzastaurin (LY317615) is a non-cyclic bisindolylmaleimide that competitively inhibits the ATP binding site of PKC-β, resulting in the inhibition of its kinase activity.[9] Its specificity toward PKC-β is relatively high with an IC50 of 0.069 *µ*M and is orally absorbed. Early pre-clinical studies have shown that enzastaurin is active against lung cancer[10–11] [both small cell lung cancer (SCLC) and NSCLC], breast cancer,[12] colon and renal cancers,[13] hepatocellular carcinoma[14] and glioblastoma multiforme[15] cell lines and tumor xenografts. We have previously shown that enzastaurin inhibits the cell growth at 5 *µ*M in mesothelioma cell lines.[16] Thus, it will be important to investigate enzastaurin’s mode of action in malignant mesothelioma to extend our understanding of PKC-β in carcinogenesis, functional association with VEGFR and to optimize the activity of enzastaurin in preclinical models. In this MPM study, we also utilized a novel quinazoline urea derivative, KRN633, which strongly and selectively inhibits VEGFR-2 tyrosine kinase activity. To examine the functional effects of enzastaurin on PKC-β, we used *C. elegans* as a model organism that has several attractive features for the study of human cancer: (a) a fully sequenced genome, (b) rather small in size (1 mm), easy to propagate with a generation time (4 days) and (c) invariant cell lineage and amenability to classical and reverse genetics using RNA interference technology.[17] We have previously shown that human lung cancer specific c-Met mutant transgenic *C. elegans* (transgenic animals) suffered from an abnormal vulval development, vulval hyperplasia and lower fecundity that are exaggerated upon addition of nicotine to the culture medium.[18] This suggested that the simple soil nematode can be used as a model organism to study cancer and for high throughput screening of potential chemotherapeutic drugs. Here, we have further substantiated the above concept by demonstrating the effect of enzastaurin on body morphology, development and behavior in *C. elegans*. We have tested the hypothesis that simultaneous treatment with KRN633 and enzastaurin might synergistically enhance their antitumor efficacies against MPM. Collectively, our results demonstrate that VEGF/VEGFR signaling axis is highly active in MPM as evidenced not only from elevated levels of VEGFR-2 and its downstream target PKC-β1 and -β2, but also based on the ability of VEGFR-2 mediated tyrosine phosphorylation and other signaling partners such as AKT. Enzastaurin appears to be very effective both at the biochemical level in suppressing phosphorylation of PKC and its target AKT and also at the cellular level as evidenced from survival assay. Compared to KRN633 that targets surface VEGFR-2, enzastaurin that inhibits an important common receptor tyrosine kinase (RTK) signaling mediator, PKC, is highly effective in suppressing MPM cell survival, which is supported by a drastic decrease in protein phosphorylation. In addition, using the *C. elegans* model, we show here that enzastaurin treatment results in changes in morphology and locomotion, and egg laying pattern, which can be used in a high throughput screening assay for future therapeutics.

## MATERIALS AND METHODS

### Immunohistochemistry and tissue microarrays

Forty-two tumor samples of MPM including 29 epithelioid (EPI, 69%) and 9 sarcomatoid (SAR, 21%) were processed into a tissue microarray (TMA) under an institutional review board approved protocol. For control, we used 10 uninvolved lung and pleura tissues with normal lung parenchyma, fibrotic pleura, giant cell reaction, and reactive mesothelium morphologies. Paraffin-embedded, formalin-fixed TMA sections were deparaffinized by two xylene rinses followed by two rinses with 100% ethanol. Antigen retrieval was performed by heating the slides in a pressure cooker filled with 7.5 mM sodium citrate (pH 6.0) or ethylenediaminetetraacetic acid (EDTA) buffer (pH 9.0). After rinsing briefly in 2× Tris-buffered saline (TBS) at pH 8, the slides were incubated for 30 minutes in 3% hydrogen peroxide in methanol to block endogenous peroxidase activity. The slides were then incubated with 0.3% bovine serum albumin in 1× TBS for 30 minutes at room temperature to reduce nonspecific background staining and then subjected to washes in 1× TBS, 1× TBS containing 0.01% Triton, and then in 1× TBS, each for 2 minutes duration. The slides were incubated for 1 hour at room temperature with mouse PKC-β1 monoclonal antibody (clone E3, Santa Cruz, CA, USA, 1:50), mouse PKC-β2 monoclonal antibody (clone 28, GeneTex, Irvine, CA, USA 1:100), rabbit VEGF polyclonal antibody (Santa Cruz, 1:100), rabbit VEGFR-2 (KDR) polyclonal antibody (Calbiochem, San Diego, CA, USA 1:100) or rabbit phospho-AKT polyclonal antibody (Abcam, Boston, MA, USA 1:100). Slides were rinsed in TBS and incubated for 30 minutes with goat anti-mouse or anti-rabbit IgG conjugated to a horseradish peroxidase-labeled polymer (Envision+ System, DAKO, Carpinteria, CA, USA). This incubation was followed by TBS rinses, visualization with diaminobenzidine chromogen (DAKO), and then counterstained with hematoxylin. Appropriate negative controls for the immunostaining were prepared by omitting the primary antibody step and substituting it with non-immune mouse or rabbit serum.

### Scoring

First, results were analyzed manually and scored for intensity as 0 (negative), 1+ (weak), 2+ (moderate), and 3+ (strong). In addition, we subjected TMA to an automated quantification by using the Automated Cellular Imaging System (ACIS) from Clarient (San Juan Capistrano, CA, USA) as previously described.[16] ACIS consists of a bright field microscope with several objectives, digital camera, an automated slide loading system, and a computer. The measurement of intensity of the staining is based on three related color parameters: the color defined by hue, the “darkness” defined as luminosity, and density of the color defined as the saturation. ACIS software was programmed by experienced user-pathologist (M.T.), by setting the color-specific thresholds, to determine the intensity of brown positivity of cells within the outlined areas of interest. For each TMA core, we selected representative areas of tumor containing comparable numbers of cells, approximately 50–100 cells. The ACIS software calculated the average intensity for each region as a measure of integrated optical density (IOD) in the nuclear compartment. The IOD of each image (region) is given as the average of optical densities of each molecule (pixel) within the region. Computing of IOD is directly proportional to the concentration of molecule recognized by the stain according to the Beer–Lambert law. IOD is a proxy for antigen content and it is calculated as intensity multiplied by brown area (in microns). For comparison purposes, we normalized the IOD value to the entire measured area by calculating IOD/10 mm^2^. IOD measurements were translated into conventional scale of negative, weak, moderate and strong staining, and compared to manual scores with strong positive correlation ranging from *r* = 0.78 to *r* = 0.93 for different markers. For final statistical analyses, we used ACIS-derived IOD measurements on four-tier scale of negative, weak, moderate and strong staining positivity.[16]

### Cell lines and tissue specimens

H2452 (SAR), H2691 (EPI), H2461 (EPI), H513 (EPI), H2596 (SAR), H2373 (SAR), H28 (SAR), MSTO-211H (biphasic), H2052 (SAR), non-malignant mesothelial cells (Met5A) and human umbilical vein endothelial cells (HUVEC) were obtained as previously described and cultured as described.[16] The Institutional Review Board at the University of Chicago approved use of human materials in this study.

### Maintenance of the nematode strains, culture conditions and synchronization of *C. elegans*

Wild type *C. elegans* N2 was obtained from the Caenorhabditis Genetics Center, University of Minnesota, St. Paul, MN, USA. The worms were grown on standard nematode growth medium (NGM) seeded with *Escherichia coli* strain OP50 as a food source at 20°C. The worms were synchronized by alkaline hypochlorite method as previously described.[17,28] L1 larvae were harvested 6 hours after feeding, L2 larvae at 20 hours, L3 larvae at 29 hours, L4 larvae at 40 hours, young adult nematodes at 52 hours, and egg-laying adult animals were obtained at 78 hours.

### Enzastaurin exposure of *C. elegans* and screening phenotypes

Enzastaurin response experiments were performed in M9 buffer (liquid culture media for the *C. elegans* experiment) as previously reported for nicotine.[19] To examine the enzastaurin dose response, individual young adult hermaphrodites were placed in six-well microtiter plates in M9 buffer at different concentrations of enzastaurin. Following 4 hour incubation with enzastaurin (10 *µ*M) medium plates at 20°C, total numbers of eggs laid by each individual animal were counted. Enzastaurin exposed animals were individually cloned and allowed to grow at 20°C and their progeny were observed under dissecting microscope. Animals in the progeny harboring a visible phenotype such as vulva defect, body morphology change, locomotion defect, and paralysis; and animals showing hyperactivity were isolated, cloned individually and examined with dissecting microscope. For detailed analysis of the phenotype, individual animals were mounted on an agar pad and observed under a Nikon Eclipse E600 fluorescence microscope equipped for Nomarski optics. Images were taken by a Hamamatsu Photonics CCD camera, and processed using the Metamorph digital imaging software.[20]

### Immunoblot analysis

Cells were exposed to varying concentrations of enzastaurin for the indicated time periods as described above. The cells were washed twice with ice-cold phosphate buffered saline (PBS), and whole cell lysates were prepared in RIPA lysis buffer [50 mM Tris (pH 8.0), 150 mM NaCl, 10% glycerol, 1% NP-40, 0.5% sodium deoxycholate, 0.1% SDS and 0.42% NaF containing protease inhibitor cocktail (1 mM phenylmethylsulfonyl fluoride, 1 mM Na_3_VO_4_, 5 mg/ml leupeptin)]. The cell lysates were cleared by centrifugation at 14,000 *g* for 15 minutes. The protein concentrations were determined by using the Bradford assay. Protein lysates (60–80 *µ*g) were separated by 7.5% sodium dodecyl sulfate polyacrylamide gel electrophoresis (SDS-PAGE) under reducing conditions and transferred to PVDF membranes (Millipore, Bedford, MA, USA). The membranes were blocked in 5% non-fat dried milk freshly made in Tris-Buffered Saline Tween-20 (TBST). Proteins were detected by immunoblotting using an enhanced chemiluminescense kit (NEN Life Science Products, Boston, MA, USA) according to manufacturer’s protocol.

### Statistical analysis

For comparisons of frequencies among different categories, the Fischer’s exact test was used.

## RESULTS

### Expression of VEGFR-2 and PKC-β1 and PKC-β2 in mesothelioma cell lines and tumor tissues

**Figure 1:**
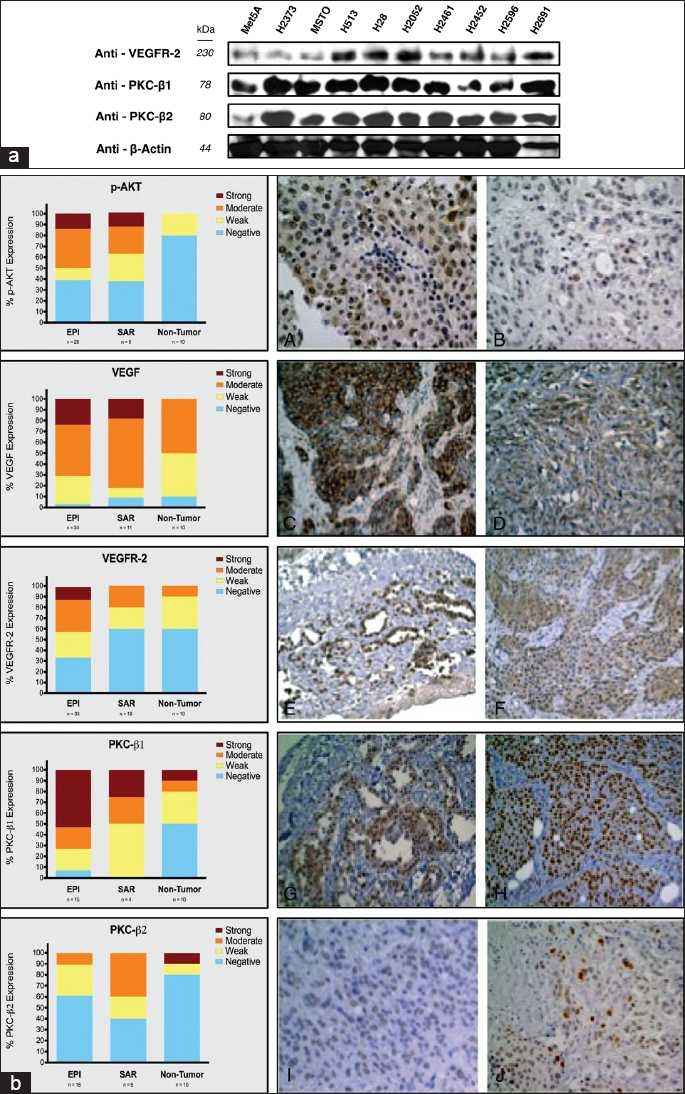
Expression of VEGF/VEGFR-2 and some of its key downstream target proteins in mesothelioma. (a) Expression levels of VEGFR-2 and PKC-β1 and PKC-β2 in mesothelioma cell lines as determined by immunoblotting. PKC-β1 and PKC-β2 showed similar levels of expression among all cell lines. However, Met5A (non-malignant mesothelial cells) expressed reduced amounts of VEGFR-2 and PKC-β2 protein. (b) Left panels show the percentage expression of p–AKT, VEGF, VEGFR-2, PKC-β1 and PKC-β2 in epithelioid (EPI), sarcomatoid (SAR) and non-tumor tissue microarray specimens and the representative images on the right panels. Right panels: panels A (strong) and B (weak) show p-AKT nuclear expression in EPI malignant pleural mesothelioma (MPM). Panels C (strong) and D (moderate) show VEGF cytoplasmic expression in EPI and SAR MPM, respectively. Panels E (strong) and F (moderate) show relative expression of VEGFR-2 in EPI MPM. Panels G (moderate) and H (strong) highlight PKC-β1 expression and panels I (weak) and J (moderate) represent PKC-β2 nuclear expression in EPI MPM tissue samples.

Since both VEGFR-2 and PKC-β isoforms, PKC-β1 and PKC-β2, play an important role in neoangeogenesis, we determined their protein expression in mesothelial cell lines [Figure 1a]. H2452 (SAR), H2691 (EPI), H2461 (EPI), H513 (EPI), H2596 (SAR), H2373 (SAR), H28 (SAR), MSTO-211H (biphasic), H2052 (SAR) and non-malignant mesothelial cells (Met5A) expressed similar amounts of PKC-β1 and PKC-β2. However, in general, the relative expression levels of VEGFR-2 and PKC-β1 and PKC-β2 proteins were higher in the various MPM cell lines compared to the non-malignant Met5A cells.

### Expression of VEGFR-2 and some of its key signaling molecules in archival malignant mesothelioma tumor tissues

As shown in Figure 1b, both VEGF and VEGFR-2 were highly expressed in EPI and SAR forms of mesothelioma and so was PKC-β1 but not PKC-β2. In contrast, strong expression of PKC-β2 is seen in less than 15% of normal tissue samples. Interestingly, the frequency of strong expression for VEGF, VEGFR-2 and PKC-β1 is much higher in EPI samples compared to SAR samples. The protein expression patterns suggested that at a cellular level, the VEGFR-2 and PKC-β1 and PKC-β2 are co-expressed and there is an increased phosphorylation of the substrate AKT in the malignant lung tissue [Figure 1b]. Colored bars represent various levels of protein expression: strong (dark brown), moderate (orange), weak (yellow), and negative (blue). Both PKC-β1 and PKC-β2 showed strong to moderate expression in SAR and EPI histologies. However, lesser expression of PKC-β2 was noted in EPI tissue. VEGF and VEGFR-2 expression was strong in EPI and VEGFR-2 expression was moderate in SAR tissues as compared to the control tissue. EPI tissue displayed high expression of PKC-β1 and VEGF, VEGFR-2 and p-AKT. SAR tissue showed higher expression of p-AKT, VEGF, and PKC-β1. The data suggested a complex signaling pattern depending on the stage of mesothelioma as evident by the expression levels of PKC-β1, PKC-β2, VEGF, and VEGFR-2.

### Antiproliferative activity of enzastaurin

**Figure 2:**
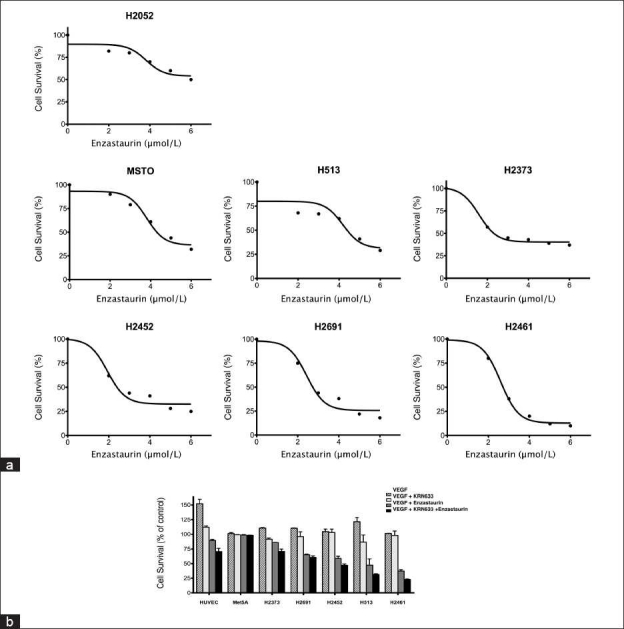
Effect of inhibition of VEGFR and its downstream target PKC on mesothelioma cell viability. (a) Antiproliferative effect of enzastaurin (1–6 µM) on a panel of malignant mesothelioma cell lines. H2052 shows high resistance to enzastaurin and H2461 is the most sensitive of all the cell lines. (b) Effect of VEGFR inhibitor, KRN633 (1 µg/ml), and enzastaurin (2.5 µM) combination on antiproliferative activity in normal and malignant mesothelioma cell lines. Human Umbilical Vein Endothelial Cells (HUVEC) response to rhVEGF is shown as a positive control. H2461, H513 and H2452 as shown above had the least cell survival among all cell lines and showed statistically significant results.

We examined the role of enzastaurin, an inhibitor of PKC-β[9] in normal mesothelial cells (Met5A), and mesothelioma cell lines [Figure 2a]. The antiproliferative effect of enzastaurin (1–6 *µ*M) was dose dependent. The Met5A control cells showed a robust cell survival at a wide range of enzastaurin concentrations (data not shown), as compared with H2052, MSTO, H513, H2373, H2452, H2691, and H2461 malignant mesothelioma cell lines. Of the cell lines examined, H2461 showed the highest sensitivity to enzastaurin, whereas H2052 was relatively less sensitive.

### Combined antiproliferative activity of enzastaurin and KRN633 in HUVEC, Met5A and mesothelioma cell lines

We investigated the effect of VEGFR-2 inhibitor, KRN633, and enzastaurin for their antiproliferative activity in normal and malignant cell lines. Figure 2b shows the mean ± SD of duplicate determinations of the cytotoxicity assay. HUVEC response to VEGF was included as a positive control, and the cell viability was determined by alamar blue dye assay. There was no discernable effect on the survival of Met5A mesothelial cells when treated singly or with a combination of enzastaurin and KRN633. However, the cell viability in the various mesothelioma cell lines when treated with enzastaurin and/or KRN633 was significantly lower compared to the controls [Figure 2b].

### Inhibition of VEGF induced phosphorylation in PKC-β1 and PKC-β2 expressing H2461 mesothelioma cells but not in Met5A normal mesothelial cells

**Figure 3:**
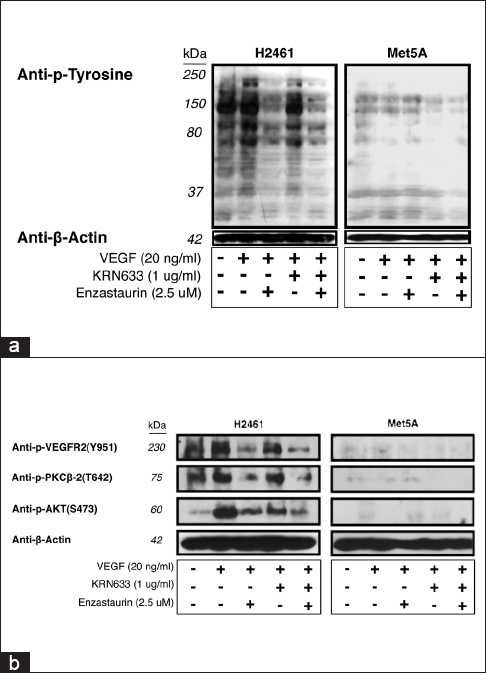
Effect of VEGFR and PKC inhibitors on VEGF induced tyrosine phosphorylation in mesothelioma cells. (a) Inhibition of VEGF-induced tyrosine phosphorylation in H2461 malignant mesothelioma cells by KRN633 and enzastaurin alone or in combination. Serum-starved mesothelioma H2461 and Met5A cells were pre-incubated with KRN633 1 µg/ml (4 hours) and/or enzastaurin 2.5 µM for 24 hours. Cells were treated with VEGF (20 ng/ml) for 20 minutes before collecting the lysates. Cell lysates were resolved by 7.5% SDS-PAGE and probed with mouse monoclonal p-Tyr antibody (Tyr 951). (b) VEGF-induced phosphorylation signaling status of VEGFR-2, PKC-β2 and AKT in the presence of KRN633 and/or enzaustarin in H2461 or Met5A cell lines.

To determine the role of VEGF induced tyrosine phosphorylation in H2461 mesothelioma cells that expressed PKC-β1 and PKC-β2, we pre-treated serum-starved mesothelioma H2461 and Met5A cells with or without the indicated concentrations of KRN633 (4 hours) and enzastaurin for 24 hours [Figure 3a]. Cells were then treated with VEGF (20 ng/ml) for 20 minutes before collecting the whole cell lysates. VEGF induced tyrosine phosphorylation was found to be inhibited by enzastaurin alone or in combination with VEGFR-2 inhibitor, KRN633, in H2461 cells.

### VEGF induced activation of signaling kinases in H2461 malignant mesothelioma cell line and inhibition of phosphorylation by KRN633 and/or enzastaurin

To assess the signaling events associated with malignant mesothelioma cells, we determined the phosphorylation status of VEGFR-2, PKC-β2, and AKT in the presence of KRN633 and/or enzastaurin in normal mesothelial Met5A cell and H2461 cells [Figure 3b]. Our results showed that in mesothelioma H2461 cell line, there was significant phosphorylation of VEGFR-2 (Y951), PKC-β2 (T642) and AKT (S473) in response to VEGF. Treatment with KRN633 or enzastaurin alone demonstrated considerable inhibition of the phosphorylation of above three proteins, and in addition, the combination of the two drugs resulted in additive inhibition in H2461 but not in Met5A cells.

### Effect of enzastaurin on the *C. elegans* phenotype

**Figure 4:**
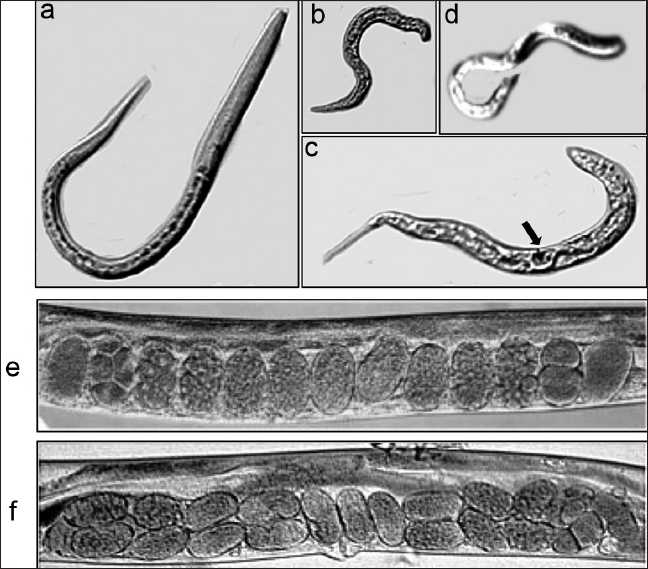
Effect of PKC inhibitor on C. elegans morphology. Altered phenotypes of C. elegans in response to enzastaurin are shown. (a) Wild-type N2 adult hermaphrodite showing normal body morphology. (b) Enzastaurin treated N2 showing defective body morphology. (c) Enzastaurin treated worms show large body vacuoles and lack of a normal vulva phenotype. (d) Induction of hyperactive movement in C. elegans by exposure to enzastaurin for few hours. (e) Arrangement of eggs in the uterus of wild-type C. elegans. (f) Altered arrangement of eggs in the uterus of enzastaurin treated wild-type C. elegans. Arrow bar highlights vacuoles of increased size in the body of enzastaurin treated worms.

An orthologue of PKC-β is expressed in *C. elegans*. We therefore determined the effect of enzastaurin treatment on development and the morphology of *C. elegans*. At lower concentrations of enzastaurin (1–5 *µ*M), there was no behavioral or morphological alterations observed in *C. elegans* larvae or adult hermaphrodites (data not shown). However, at higher concentrations, nematodes demonstrated uncoordinated hyperactivity movement and defects in body morphology [Figure 4a–d]. In addition, enzastaurin treated worms revealed abnormal arrangement of eggs compared to N2 controls [Figure 4f compared to e].

## DISCUSSION

MPM is a difficult disease to treat. Even with the best of therapies, the prognosis is quite poor.[1–2] In order to develop novel therapeutics, we investigated the role of PKC pathway that is triggered by ligation of VEGFR-2. We show here that the RTK VEGFR-2, its natural ligand VEGF, the downstream targets PKC-β1 and -β2, as well as AKT were overexpressed and/or highly active in MPM. The PKC inhibitor, enzastaurin, had significant adverse effects on the viability of a variety of MPM cell lines, which corresponds to suppression of not only PKC enzymatic activity but also that of VEGFR-2 and AKT kinase activities. In contrast, the VEGFR inhibitor, KRN633, demonstrates moderate inhibitory effects on the kinase activity of its primary target VEGFR-2. A striking observation was the dramatic decrease in VEGF induced tyrosine phosphorylation in enzastaurin treated MPM cells. The above biochemical effects parallel the effects on cellular functions such as survival and migration. The combinatorial effects of the above two drugs on MPM cell lines was at the most additive. Cancers, in general, appear to be highly addicted to growth factor signals that are reflected in not only the overexpression of the growth factors themselves but also the expression of key intracellular signaling molecules.[21–22] We, and others, have previously shown that the RTKs such as EGFR, c-Met and RON are overexpressed and are highly active in lung cancers and MPM. However, unlike the gain-of-function mutations of the above RTKs frequently seen in lung cancers,[21–25] we have not encountered any such mutations of the PKC isoforms investigated in the cohort of MPM tumor samples (data not shown). Based on the mutational and amplification analysis of *PKC*-β2 gene in MPM (9 cell lines and 33 tumor tissues), there was no amplification seen of PKC-β2; however, in two different tumor tissues, there were synonymous single nucleotide variants (SNVs), 119/T7TC: Pro and 1770C7CT: Gly. Both of these SNVs were in the ATP binding domain (data not shown).

The classification for MPM is based upon morphology and consists of EPI, biphasic, and SAR histology.[2] Our data clearly revealed that the percentage of tumor samples that show strong expression of VEGFR, its ligand VEGF and the downstream targets PKC-β1 and p-AKT is much higher in EPI compared to the SAR type of MPM. Interestingly, the expression of PKC-β2 in the MPM tumor samples revealed rather mixed results. While the MPM samples frequently revealed weak to moderate expression of PKC-β2, the strong expressors were only found in non-tumor tissues. Since PKC-β is a conventional form of PKC that requires diacyl glycerol and ionic calcium for its activation, the significance behind this subtle difference is however not clear. Based on our data, it is clear that drugs that can specifically target PKC-β are likely to be more beneficial to treat MPM and are likely to have the least side effects. The PKC-β is an indispensable downstream mediator of VEGFR signals.[26] Our results clearly demonstrate that the PKC inhibitor, enzastaurin, is more effective in killing MPM cells than the VEGFR inhibitor, KRN633. Assuming that both drugs are equally efficacious in targeting their respective kinases, the underlying mechanism behind enzastaurin efficacy could be that PKC is downstream of several RTKs including VEGFR,[27] and therefore, inhibition of PKC could in turn inhibit multiple growth factor signaling pathways that contribute to growth and survival of MPM cells. A most striking observation in the current study was the dramatic decrease in VEGF induced tyrosine phosphorylation in MPM cells treated with enzastaurin, a PKC inhibitor whose target is downstream of the RTK [Figure 3a and b]. A scenario that could explain this observation is that under normal circumstances, PKC could phosphorylate a tyrosine phosphatase, thereby inhibiting its activity. In a cell treated with enzastaurin, the above inhibition is probably relieved, resulting in unchecked tyrosine phosphatase activity that led to the observed dramatic decrease in both the specific (VEGFR-2) and the general decrease in VEGF induced tyrosine phosphorylation. In *C. elegans* nematodes, there are four isoforms of PKC, of which the PKC1 isoform is specifically expressed in neurons.[28] In order to establish the proof of principle that *C. elegans* model could be used for the study of PKC mediated signaling and for the purpose of establishing a model that is amenable for high throughput screening, we showed specific enzastaurin induced changes in overall morphology, motility and egg laying pattern. It would be important in generating transgenic worms that overexpress constitutively active form of PKC-β1 and use them for screening for potent inhibitors. Also, as we have shown previously for inducing a potential lung cancer model, *C. elegans* can be exposed to nicotine[18] or smoke or smoke products, we can potentially mimic a MPM model by exposing *C. elegans* to asbestos fibers. Chrysotile has been used more than any other type and accounts for about 95% of the asbestos found in buildings in the USA. We have preliminarily examined the toxic effect of this fiber in control (N2) *C. elegans*. *C. elegans* was cultured in M9 medium for 2 weeks with or without chrysotile fibers (asbestos), after which survival and rates of developmental defects were estimated. Chrysotile reduced survival and induced locomotion defects. It would be important now to determine the effects on the PKC (and other molecule) axis with this model and as well determine the effects of drugs such as enzastaurin.

In conclusion, we have unambiguously shown that VEGFR/PKC/AKT signaling axis is highly active in MPM and that the signals mediated by this pathway are essential for the survival of MPM. We also demonstrate here that PKC inhibitor, enzaustarin, is more effective in killing MPM cells compared to KRN633 and the combination of the two drugs is additive. Most importantly, *C. elegans* model can be used to investigate PKC signaling, especially in the context of drug screening.

## AUTHOR’S PROFILE

**Dr. Ravi Salgia,** is an expert in the area of thoracic oncology, translational research and basic science research. His main research interest is to arrive at novel targeted therapeutics to enhance the quality of life and survival for patients with cancer. He and his lab have recently identified several novel receptor tyrosine kinases that are abnormal in lung cancer. He is an expert in signal transduction, as related to growth factor receptors and oncogenes/ tumor suppressor genes, and is developing novel inhibitors based on these pathways. He is also an expert in tumor and body fluid biomarkers, and is applying this knowledge to develop new serum/tumor tissue tests for thoracic oncology.
